# *Cryptosporidium* spp. and *Enterocytozoon bieneusi* in introduced raccoons (*Procyon lotor*)—first evidence from Poland and Germany

**DOI:** 10.1007/s00436-016-5245-5

**Published:** 2016-09-14

**Authors:** Kinga Leśniańska, Agnieszka Perec-Matysiak, Joanna Hildebrand, Katarzyna Buńkowska-Gawlik, Agnieszka Piróg, Marcin Popiołek

**Affiliations:** 1 Department of Parasitology, Institute of Genetics and Microbiology, Wrocław University, Przybyszewskiego 63, 51-148 Wrocław, Poland; 2 Department of Invertebrates Systematic and Ecology, Institute of Biology, Wrocław University of Environmental and Life Sciences, Kożuchowska 5b, 51-631 Wrocław, Poland

**Keywords:** Raccoon, *Cryptosporidium* spp., *Enterocytozoon bieneusi*, Genotyping

## Abstract

The raccoon (*Procyon lotor*) carnivore native to North America is a fast spreading, invasive species in the Europe now. At the moment, the highest population occupies areas near the German-Polish border. The data on the occurrence of *Cryptosporidium* spp. and microsporidia in raccoons is limited to North America’s territory and is totally lacking in the case of their introduction to Europe. Therefore, the objective of this study was to investigate the occurrence of microparasites, i.e., *Cryptosporidium* spp. and microsporidia in the introduced raccoons obtained from localities in Poland and Germany. A PCR-based approach that permitted genetic characterization via sequence analysis was applied to raccoon fecal samples (*n* = 49), collected during 2012–2014. All fecal samples were simultaneously tested with the use of genetic markers, and DNA of microsporidia and *Cryptosporidium* spp. was detected among the examined raccoons. The results of our research confirmed the presence of *Cryptosporidium* skunk genotype and *Enterocytozoon bieneusi* NCF2 genotype. The results suggest a possible role of raccoons in the contamination of the environment, including urban areas, with pathogens of zoonotic significance as well as their role in the transmission and introduction of new genotypes of microparasites in the areas where *P. lotor* has not been observed yet. To our knowledge, there has been no literature data on the above genotypes detected previously in humans or animals from the examined study sites so far.

## Introduction

The raccoon is a North American carnivore which was introduced to Japan and Europe in the 20th century. In Europe, as a result of escaped pets, releases, and escapes from fur farms, raccoons are distributed almost across the whole mainland (Beltrán-Beck et al. 2012). A rapid expansion of this species has been observed in wild environment since the 1980s mainly on the German territory (Hohmann et al. 2001; Stubbe 1999). At present, the largest European stable population occurs in Germany (over one million individuals) (Hohmann et al. 2000; Michler and Michler 2012), but smaller populations inhabit also other European countries (Beltrán-Beck et al. 2012; Schley et al. 2001; Stubbe 1999). In Poland, the first individuals in wildlife were observed in the 1940s (Bogdanowicz and Ruprecht 1987). In the 1980s and the 1990s, a wild population was reported in Western Poland, and since that time, the abundance of the raccoons on the Polish territory has grown rapidly (Bartoszewicz et al. 2008; Bartoszewicz and Okarma 2007; Biedrzycka et al. 2014; Popiołek et al. 2011).

Raccoons, which become one of the fastest spreading wild living population, are often found in forested areas as well in urban space near human settlements, where they can find alternative sources of food but also contribute to the transmission of many zoonotic groups of parasites to other wildlife and humans (Kresta et al. 2009). Some studies have shown that species introduced into a novel environment often lose their own parasites during the course of a new population establishment (Torchin et al. 2003; Torchin and Mitchell 2004) but also encounter and accumulate parasites that occur in newly colonized areas. In addition, there may be a significant probability of raccoons introducing some new parasite species, recorded previously in individuals from North America, into European ones. The raccoon as an alien and invasive species, both wild living and potentially synanthropic, may serve as a susceptible host for opportunistic intestinal parasites. The current epidemiological data on *Cryptosporidium* spp. and microsporidia has raised public health concerns about the zoonotic nature of transmission of these microparasites. The knowledge on raccoon as reservoir hosts of the abovementioned group of parasites is rather limited and concerns Central and North Americas’ territories (Feng et al. 2007; Guo et al. 2014; Perz and Le Blancq 2001; Snyder 1988; Sulaiman et al. 2003; Zhou et al. 2004). On the other hand, there is no data on these microparasites in the case of invasive European raccoons.

*Enterocytozoon bieneusi* and *Encephalitozoon* spp. are the major microsporidians infecting humans and animals worldwide (Santin and Fayer 2011). At present, over 240 *E. bieneusi* genotypes have been identified (Matos et al. 2012; Zhao et al. 2015). By internal transcribed spacer (ITS) sequence analysis of *E. bieneusi* genotypes, eight different groups of all genotypes were established (Karim et al. 2014). A large cluster named as group 1 contains more than 94 % published genotypes of *E. bieneusi* (Henriques-Gil et al. 2010). The genotypes within this group are found both in humans and animals. Even though some genotypes are genetically similar to human pathogenic ones, they have been found only in animals so far, suggesting their zoonotic potential (Henriques-Gil et al. 2010). The remaining genogroups (2–8) are found mostly in specific hosts and wastewater (Guo et al. 2014). *Encephalitozoon* spp., another microsporidia group, has been generally studied among humans and domestic animals; there is still insufficient information on the role of wild living animals, including raccoons, which may be a potential source of zoonotic contamination with this microsporidia species.

So far, 30 species and over 100 genotypes of *Cryptosporidium* have been described in various vertebrate hosts and environmental sources (Kváč et al. 2014). Among them, *Cryptosporidium hominis* and *Cryptosporidium parvum* are responsible for over 90 % of human cryptosporidiosis cases (Rossle and Latif 2013). Wild living mammals, including carnivores, have been described as reservoirs of several *Cryptosporidium* species, especially *C. parvum* and *Cryptosporidium muris* (Fayer et al. 2010; Ryan and Hijjawi 2015) but also *Cryptosporidium meleagridis*, *Cryptosporidium ubiquitum*, *Cryptosporidium felis*, *Cryptosporidium canis*, *Cryptosporidium cuniculus*, *Cryptosporidium* skunk genotype, chipmunk genotype, and other novel genotypes (Chalmers et al. 2009, 2011; Elwin et al. 2012; Li et al. 2014; Plutzer and Karanis 2009; Robinson et al. 2008; Xiao 2010; Xiao et al. 1999).

Therefore, the aim of this preliminary study was to investigate the presence of intestinal microparasites occurring in the raccoon population in newly colonized areas of Western Poland and Germany. Molecular analyses were conducted to identify and genotype *Cryptosporidium* spp. and microsporidia species emphasizing their zoonotic potential in European raccoons.

## Materials and methods

### Study areas and collection of material

**Fig. 1:**
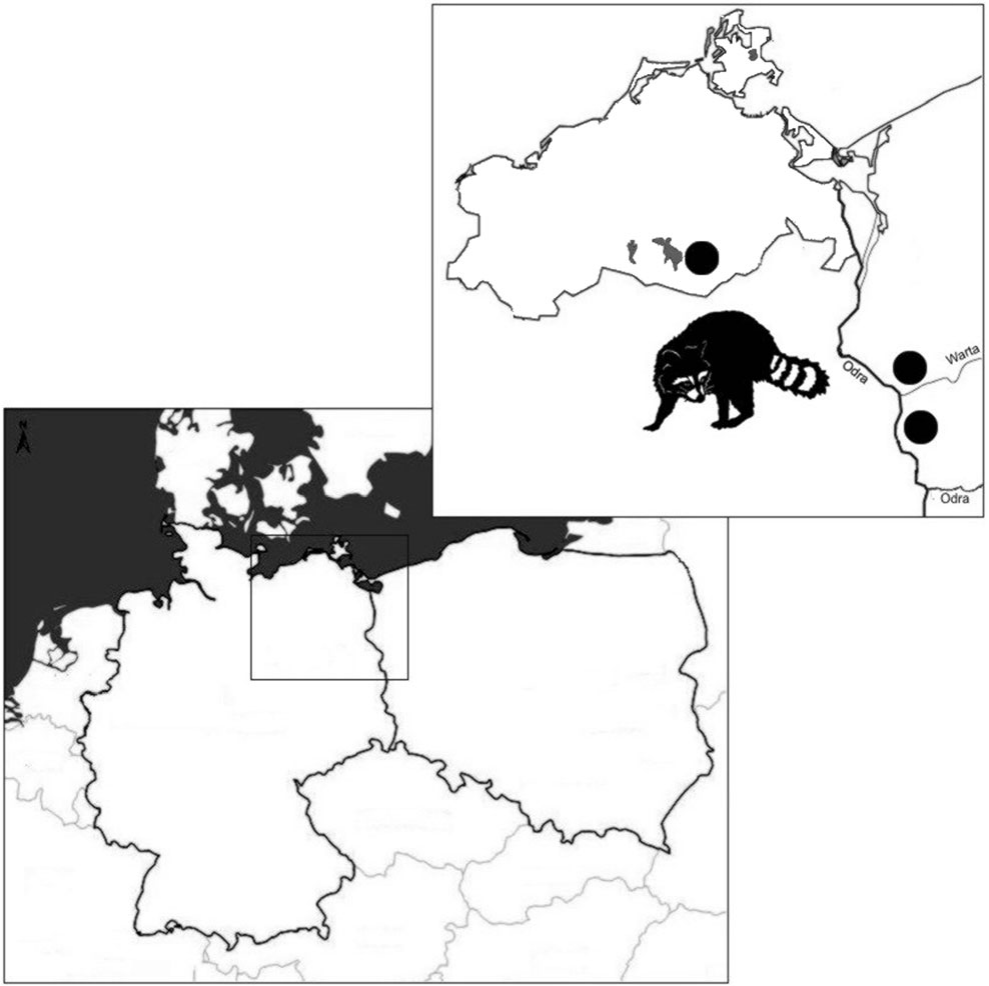
The map of Poland and Germany showing geographical origin (*black dots*) of wild raccoons obtained for this study

This study was carried out on 49 raccoons, comprising 31 males and 18 females, collected from hunters and road-kills from the area of Kostrzyn on the Oder and Warta Mouth National Park, Poland (*n* = 32), and from localities near the Müritz National Park, Mecklemburg-Vorpommern, Germany (*n* = 17) (Fig. 1). A suburban environment of the city of Kostrzyn is located in the surroundings of Warta Mouth National Park (WMNP) (52 °34′ N, 14° 43′ E) which covers about 80 km^2^ of the Warta River. Here, raccoons occupy relatively small territories and live in high density (0.7–2.5 individuals per 1 km^2^)—their home ranges overlap with an average of 80 %. This German-Polish border area is a zone of high human activity associated with traffic at petrol stations, restaurants, and hotels. WMNP is a part of Natura 2000 project, which makes it also attractive for tourists. Müritz National Park (MNP) (53° 27′ N, 12° 49′ E) located in Mecklenburg-Vorpommern contains a large amount of wetland habitats, especially bog and swamp districts. This area demonstrates a very opportune habitat for raccoon—its population density in the area is twice as much as that in the middle part of Germany (6–8 individuals per 1 km^2^) (Fischer et al. 2016; Hohmann 1998; Köhnemann and Michler 2008; Muschik et al. 2011).

Frozen carcasses were delivered to the laboratory of Department of Parasitology UWr and dissected. Collected fecal samples were kept at −20 °C for further analysis. Each animal was used for only one fecal specimen.

### DNA extraction and PCR amplification

DNA was isolated from all 49 fecal samples using GeneMATRIX Stool DNA Purification Kit (EURx, Gdańsk, Poland) according to the manufacturer’s instructions. Obtained DNA was stored at −20 °C until further use.

PCR amplification was performed on a sets of nested primers amplifying the ITS region of the ribosomal ribonucleic acid (rRNA) gene, i.e., EBITS3, EBITS4 and EBITS1, EBITS2.4 for *E. bieneusi* (Buckholt et al. 2002) and INT580F, INT580R and Msp3, Msp4a for *Encephalitozoon* spp. (Katzwinkel-Wladarsch et al. 1996). A fragment of *Cryptosporidium* 18S rRNA and *Cryptosporidium* oocyst wall protein (COWP) genes were amplified (Pedraza-Diaz et al. 2001; Spano et al. 1997; Xiao et al. 1999). For the amplification of actin genes, we used cycling parameters elaborated by Sulaiman et al. (2002). For all PCR reactions, negative and positive controls were performed with sterile water and reference DNA, respectively. Secondary PCR products were subjected to electrophoresis on a 1.0 % agarose gel and stained with Midori Green (Nippon Genetics Europe GmbH). Products of expected size were purified using QIAquick PCR Purification Kit (Qiagen, Hilden, Germany) and stored at 4 °C until sequencing.

### Nucleotide sequencing

Products were sequenced in both directions on Applied Biosystems ABI PRISM 3100-Avant Sequencer (SEQme, the Czech Republic). The nucleotide sequences obtained in this study were edited using DNA Baser Sequence Assembly software (Heracle BioSoft SRL Romania) then aligned with reference sequences of *Cryptosporidium* spp. and *E. bieneusi* available in GenBank. Phylogenetic analyses were performed using MEGA6 software (Tamura et al. 2013). Trees were inferred by neighbor joining (NJ) method based on the Kimura 2-parameter distance model; bootstrapping was performed using 1000 replicates. Sequences from this study have been deposited in GenBank database under the accession numbers KX639723 and KX621279.

### Statistical analysis

Prevalence was expressed as a ratio of a number of PCR positive samples for *Cryptosporidium* spp. 18S rRNA or/and COWP genes and the total number of examined samples. Contingency tables were used to compare prevalence between the sex of the raccoons and the different sampling areas using the chi-square test; *p* < 0.05 was considered statistically significant (STATISTICA®12).

## Results

Nested PCR detected *E. bieneusi* in 2 of 49 (4.1 %) examined fecal samples of raccoons. Both positive samples, one obtained for a female and the other one for a male raccoon, were recorded from the area of Poland. The overall prevalence of *Cryptosporidium* spp. was estimated in 34.7 % (17/49) with infection rates of 38.9 % (7/18) and 32.3 % (10/31) observed in female and male raccoons, respectively. The prevalence of parasites according to the study sites was determined in 43.8 % (14/32) and 17.6 % (3/17) for Poland and Germany, respectively. No statistically significant differences were found in the occurrence of *Cryptosporidium* spp. and *E. bieneusi* between the sampling areas and the sex of the examined raccoons. In our survey, we did not detect any DNA of *Encephalitozoon* spp. in raccoons.

**Fig. 2:**
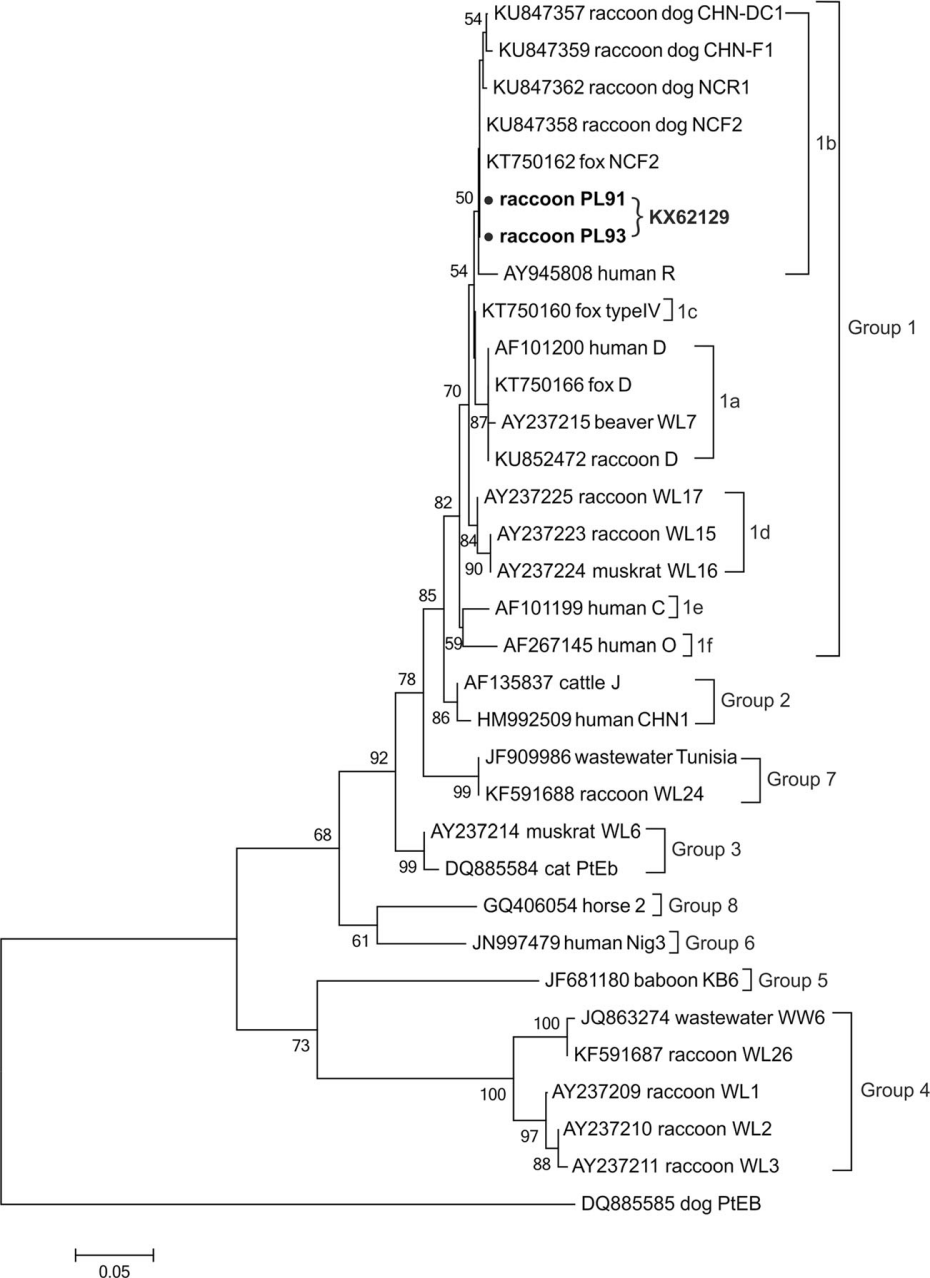
The phylogenetic relationship of *Enterocytozoon bieneusi* genotypes identified in the present study (indicated by *solid circles*) and others as inferred by a neighbor-joining analysis of ITS sequences. Bootstrapping was performed using 1000 replicates, and the values below 50 % are not shown. The *E. bieneusi* group terminology is based on the works of Guo et al. (2014), Zhao et al. (2015), and Xu et al. (2016)

The analysis of the ITS region of *E. bieneusi* revealed the existence of one known genotype in both positive samples, namely NCF2. The phylogenetic analysis showed that the genotype was identical to the ones previously reported in fox (KT750163) and raccoon dog (KU847358) in China and clustered into group 1 (Fig. 2).

**Fig. 3:**
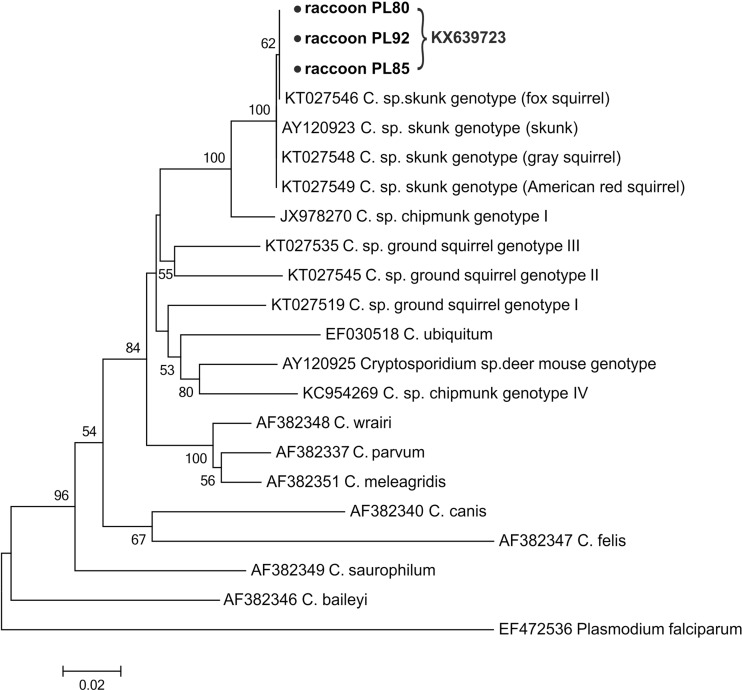
The phylogenetic relationship of *Cryptosporidium* sp. skunk genotype identified in present study (indicated by *solid circles*) and others as inferred by a neighbor-joining analysis of the actin gene sequences. Bootstrapping was performed using 1000 replicates, and the values below 50 % are not shown

The isolates from *Cryptosporidium* positive samples obtained from the amplification of 18S rRNA and/or COWP genes were genotyped by the sequence analysis of the actin gene. The only *Cryptosporidium* genotype, namely skunk genotype (identified from sequences of the actin gene), was detected in 9 out of 14 actin positive raccoons. Although the obtained sequences were not of the same length, they were identical to the isolate obtained from Eastern fox squirrel (KT027546) (Fig. 3). Isolates from the remaining actin positive raccoons yielded sequences of insufficient quality to include in the analyses.

## Discussion

In this study, we have molecularly identified the presence of *E. bieneusi* NCF2 genotype and *Cryptosporidium* skunk genotype in the Polish-German population of introduced raccoons. To our knowledge, this has been the first report on these groups of parasites in raccoons colonizing Europe. By now, literature data concerning this issue is based on the reports from the area of North America, where raccoon represents the native fauna. Also there are no available studies on the parasites in the population of raccoons introduced to Japan, but there are surveys on the raccoons introduced to Europe and Japan that concern some other bacterial and viral pathogens as well as parasites of protozoan and helminth species (Bauer 2013; Beltrán-Beck et al. 2012; Popiołek et al. 2011).

Our survey has shown that 2 out of 49 fecal samples of raccoons were *E. bieneusi* positive. During the phylogeny analysis, the detected genotype NCF2, clustered with other genotypes of group 1, suggesting its zoonotic potential. This newly identified *E. bieneusi* NCF2 genotype in European raccoons has so far been present only in farmed foxes and raccoon dogs from China (Xu et al. 2016; Zhang et al. 2016). The studies undertaken by Guo et al. (2014) showed that the infection rate of *E. bieneusi* in raccoons from a New York watershed area was as high as 82 % (18/22). The molecular studies conducted by Sulaiman et al. (2003) and Guo et al. (2014) revealed the presence of human pathogenic genotypes i.e., Peru 11, EbpC, WL15, and D genotypes. Additionally, the following raccoon-adapted genotypes were identified WL1-3, WL13, WL15-17, WL24, WL26, and WW6 (Guo et al. 2014; Sulaiman et al. 2003).

*Cryptosporidium* infection has been reported in raccoons (Carlson and Nielsen 1982; Snyder 1988; Zhou et al. 2004; Ziegler et al. 2007). Using indirect immunofluorescent assay, Snyder (1988) found that 13 % of wild raccoons were infected. By using molecular tools, *C. parvum* infection was found in 1 of 5 (20 %) raccoons from wildlife parks in New York State (Perz and Le Blancq 2001), and 2 of 51 (3.9 %) raccoons were *Cryptosporidium* skunk genotype positive in wetlands adjacent to the Chesapeake Bay (Zhou et al. 2004). *Cryptosporidium* skunk genotype and *C. ubiquitum* were identified in the raccoons and storm water from a New York watershed area (Feng et al. 2007). The *Cryptosporidium* skunk genotype was detected also in Eastern gray, American red and fox squirrels, river otters, and striped skunks from the area of the USA (Feng et al. 2007; Stenger et al. 2015; Ziegler et al. 2007). The phylogenetic analysis showed that the isolates obtained in the present study were organized in skunk genotype clade containing isolates previously identified among squirrels and skunk. Although uncommon, human infections with skunk genotype have also been reported (Davies et al. 2009; Elwin et al. 2012; Robinson et al. 2008). The wildlife origin of genotypes is increasingly recognized as an important environmental source of *Cryptosporidium* infection in humans, and shifting boundaries between wildlife and humans could result in the emergence of novel pathogens (Stenger et al. 2015). Considering that more than 75 % of human cases of cryptosporidiosis are determined to have the zoonotic origin and are related to wildlife and domestic animals, studies concerning a wide range of wild living hosts seem to be reasonable.

Despite the fact that the raccoon as an alien species has been present in Europe for 80 years, the knowledge about its parasitofauna is still insufficient, especially concerning microparasites. As our results have shown, raccoon as an introduced species lost many of its originally detected *Cryptosporidium* spp. and *E. bieneusi* genotypes. On the other hand, it encountered the *E. bieneusi* genotype which has not been identified either in Europe or in the North America. We suppose that *E. bieneusi* NCF2 genotype might be of the raccoon dog origin which is another species introduced from the area of East Asia. *Cryptosporidium* skunk genotype has been detected for the first time in the examined areas suggesting a North American origin. Thus, our results have shown that introduced raccoons could be considered as a potential source of human pathogenic *Cryptosporidium* skunk genotype and potentially pathogenic *E. bieneusi* NCF2 genotype.
